# Efficient Arsenic Metabolism — The *AS3MT* Haplotype Is Associated with DNA Methylation and Expression of Multiple Genes Around *AS3MT*

**DOI:** 10.1371/journal.pone.0053732

**Published:** 2013-01-14

**Authors:** Karin S. Engström, Mohammad Bakhtiar Hossain, Martin Lauss, Sultan Ahmed, Rubhana Raqib, Marie Vahter, Karin Broberg

**Affiliations:** 1 Section of Occupational and Environmental Medicine, Department of Laboratory Medicine, Lund University, Lund, Sweden; 2 Section for Metals and Health, Institute of Environmental Medicine, Karolinska Institutet, Stockholm, Sweden; 3 International Centre for Diarrhoeal Disease Research, Bangladesh (ICDDR,B), Dhaka, Dhaka, Bangladesh; 4 Department of Oncology, Lund University, Lund, Sweden; Geisel School of Medicine at Dartmouth, United States of America

## Abstract

Arsenic is a very potent toxicant. One major susceptibility factor for arsenic-related toxicity is the efficiency of arsenic metabolism. The efficiency, in turn, is associated with non-coding single nucleotide polymorphisms (SNPs) in the arsenic methyltransferase *AS3MT* on chromosome 10q24. However, the mechanism of action for these SNPs is not yet clarified. Here, we assessed the influence of genetic variation in *AS3MT* on DNA methylation and gene expression within 10q24, in people exposed to arsenic in drinking water. DNA was extracted from peripheral blood from women in the Argentinean Andes (N = 103) and from cord blood from new-borns in Bangladesh (N = 127). *AS3MT* SNPs were analyzed with Sequenom or Taqman assays. Whole genome epigenetic analysis with Infinium HumanMethylation450 BeadChip was performed on bisulphite-treated DNA. Whole genome gene expression analysis was performed with Illumina DirectHyb HumanHT-12 v4.0 on RNA from peripheral blood. Arsenic exposure was assessed by HPLC-ICPMS. In the Argentinean women, the major *AS3MT* haplotype, associated with more efficient arsenic metabolism, showed increased methylation of *AS3MT* (p = 10^−6^) and also differential methylation of several other genes within about 800 kilobasepairs: *CNNM2* (p<10^−16^), *NT5C2* (p<10^−16^), *C10orf26* (p = 10^−8^), *USMG5* (p = 10^−5^), *TRIM8* (p = 10^−4^), and *CALHM2* (p = 0.038) (adjusted for multiple comparisons). Similar, but weaker, associations between *AS3MT* haplotype and DNA methylation in 10q24 were observed in cord blood (Bangladesh). The haplotype-associated altered CpG methylation was correlated with reduced expression of *AS3MT* and *CNNM2* (r_s_ = −0.22 to −0.54), and with increased expression of *NT5C2* and *USMG5* (r_s_ = 0.25 to 0.58). Taking other possibly influential variables into account in multivariable linear models did only to a minor extent alter the strength of the associations. In conclusion, the *AS3MT* haplotype status strongly predicted DNA methylation and gene expression of *AS3MT* as well as several genes in 10q24. This raises the possibility that several genes in this region are important for arsenic metabolism.

## Introduction

Arsenic is a widespread environmental pollutant with strong adverse effects in humans, including increased incidence of cancer and effects on skin, respiratory tract, liver, and immune function [1], [2], [3], [4], [5]. Inorganic arsenic is efficiently absorbed in the gastrointestinal tract and is metabolized in the body by a series of reduction and methylation reactions, producing methylarsonic acid (MMA) and dimethylarsinic acid (DMA), both of which are excreted in the urine [6]. In humans, efficient methylation from inorganic arsenic to DMA is associated with a high rate of arsenic excretion in the urine [6]. Incomplete arsenic metabolism, with higher urinary inorganic arsenic and MMA, but lower DMA, seems to be a marker of increased susceptibility to arsenic-related diseases, including cancer [7], [8].

The main methyltransferase in arsenic metabolism is arsenic (+3 oxidation state) methyltransferase (AS3MT), which can methylate both inorganic arsenic and MMA [9], although its affinity for these two substrates may differ. The *AS3MT* gene is located in chromosome band 10q24, contains 11 exons and spans 32 kilobases [10]. Real time-polymerase chain reaction (PCR) analysis of mature rat tissues detected *AS3MT* expression in the heart, adrenal gland, urinary bladder, brain, kidney, lung, and liver [9].

Different human populations have varying efficiencies of arsenic metabolism and these efficiencies may reflect underlying genetic differences. For example, compared to other populations, indigenous populations in the Andes demonstrate a uniquely proficient arsenic metabolism, with low urinary excretion of MMA and high excretion of DMA [11]. Moreover, this efficient-metabolizing phenotype was recently shown to be determined by genetic factors; eight single nucleotide polymorphisms (SNPs) (seven non-coding) in *AS3MT* had strong effects on arsenic metabolism in the population living in the Argentinean Andean highlands, and the efficient-metabolizing haplotype was very common (70%) [12]. Six of these SNPs were also shown to influence arsenic metabolism in people in Bangladesh [12], but the haplotype frequency was much lower (17%) than that in the Argentinean population. All but one of the SNPs in the *AS3MT* efficient-metabolizing haplotype are non-coding; two of the non-coding SNPs were shown to decrease the expression of *AS3MT* [12]. Still, the mechanisms for alteration of gene expression are unknown; possibly the haplotype is associated with epigenetic changes in DNA methylation that in turn affects gene expression. The aim of the present study was to assess the influence of genetic variation in *AS3MT* on DNA methylation and gene expression within 10q24. This work was performed in two distinct human populations, in a subgroup from the above-mentioned population from the Argentinean Andean highlands [12] and a mother-child cohort from Bangladesh.

## Results

### Background Data, *AS3MT* SNPs in CpG Sites and Influence on *AS3MT* Methylation

The characteristics of the different study groups in Argentina and Bangladesh are described in Table 1. In Argentina, only women were analyzed (N  = 94 for DNA methylation; N  = 90 for gene expression) and their median age was 32 years, they had a median BMI of 24 and 42% reported that they chew coca leaves. Their median total urinary arsenic was 188 µg/L (5/95 percentile 16/620). Their characteristics were similar to the full study population (N  = 172) (Table 1). In Bangladesh both newborn boys (N  = 62) and girls (N  = 65) were examined. Their mothers had a median age of 25, BMI of 20 and their median total arsenic in urine was 68 µg/L (5/95 percentile 20/460).

**Table 1:** Characteristics of the study subjects in Argentina and Bangladesh.

Argentina	DNA methylation Argentina (N = 94)		Gene expression (N = 90)		*AS3MT* 3′UTR expression (N = 55)		Full study pop. (N = 172)	
	Median	5/95	Median	5/95	Median	5/95	Median	5/95
Age (years) - All	32	17/57	32	15/61	32	15/65	34	17/63
- 0/1/2 haplotype copies*	33/31/32							
p-value†	0.93							
BMI (kg/m^2^) - All	24	18/33	24	18/33	24	18/33	24	19/35
- 0/1/2 haplotype copies	25/23/24							
p-value	0.46							
Chewing coca leaves - All	42% users		43% users		44% users		46% users	
- 0/1/2 haplotype copies	57/36/44							
p-value	0.53							
Total urine As (µg/L)‡ - AAll	188	16/620	203	16/622	268	136/659	205	20/500
- 0/1/2 haplotype copies	267/197/178							
p-value	0.63							
Bangladesh	(N = 127)		NA§		NA		NA	
Sex (% female) - All	51							
- 0/1/2 haplotype copies	50/50/80							
p-value	0.42							
Birth weight (kg) - All	2.7	2.2/3.3						
- 0/1/2 haplotype copies	2.8/2.6/2.6							
p-value	0.15							
Total urine As (µg/L)‡–All	68	20/460						
- 0/1/2 haplotype copies	63/110/75							
p-value	0.42							
Mothers age - All	25	17/36						
- 0/1/2 haplotype copies	25/23/25							
p-value	0.14							
Mothers BMI - All	20	17/26						
- 0/1/2 haplotype copies	20/20/20							
p-value	0.63							
Gestational age - All	39	36/42						
- 0/1/2 haplotype copies	39/38/39							
p-value	0.14							
Asset score - All	1.3	−4.2/3.4						
- 0/1/2 haplotype copies	1.3/1.2/−0.8							
p-value	0.50							

For comparison, also the full study population in Argentina [12] is included.

* Median stratified for haplotype. N = 7 (0 copies), 41 (1 copy), and 45 (2 copies) in Argentina (one individual missing haplotype data), N = 90 (0 copies), 32 (1 copy), 5 (2 copies) in Bangladesh, and N = 17 (0 copies), 71 (1 copy), and 84 (2 copies) in the full study population in Argentina.

† P-values from non-parametric Kruskal-Wallis test, numbers of haplotype copies is the grouping variable.

‡ Adjusted for specific weight. Measured at gestational week 6–8 in Bangladesh.

§ NA =  not applicable, since no RNA was available for analysis in the Bangladeshi study population.

We next examined the frequencies of the *AS3MT* haplotype in these two populations and found them to be 69% in Argentina (9% with 0 copies, 44% with 1 copy and 47% with 2 copies – estimated from eight SNPs) and 17% in Bangladesh (71% with 0 copies, 25% with 1 copy and 4% with 2 copies – estimated from five SNPs). The *AS3MT* haplotype appeared to have a strong effect on the arsenic metabolite pattern in urine, an example in the Argentinean women: increasing copies of the *AS3MT* haplotype were associated with a higher percentage of DMA (medians 73.0% for 0 copies, 76.0% for 1 copy, and 83.4% for 2 copies; p<0.001 in a general linear model adjusted for U-As). There were no statistically significant differences between the haplotypes in terms of total urinary arsenic (marker of arsenic exposure), age, BMI, and chewing of coca leaves (evaluated in the Argentinean population), or in terms of arsenic exposure during different points during gestation, measured in maternal urine around gestational weeks 6–8 or 30, mothers’ age, mothers’ BMI, asset score (similar to socioeconomic status), sex of the baby and gestational age (evaluated in the Bangladeshi population) (Table 1).

One potential effect of SNPs on DNA methylation may come from sequence changes that introduce or remove methylation sites; therefore we examined the SNPs associated with the *AS3MT* haplotype to determine whether these SNPs altered methylation sites and whether these sites were in CpG islands or shores. In the Argentinean women with the major *AS3MT* haplotype (constituted by rs7085104 G, rs3740400 C, rs3740393 C, rs3740390 A, rs11191439 T, rs11191453 C, rs10748835 A, and rs1046778 C), seven out of the eight *AS3MT* SNPs are non-coding [12]. The non-coding rs7085104 (5′ of *AS3MT*) and rs3740400 (intron 1) and the non-synonymous rs11191439 (MetThr exchange at exon 6) SNPs result in new CpG sites (Table S1). The rs7085104 G- and rs3740400 C-alleles introduce CpG sites in the 5′region and the rs11191439 T-allele removes a CpG site. Rs7085104 is situated in a CpG shore 181 basepairs (bp) 5′ of a 424 bp long CpG island, in which rs3740400 is situated. Rs11191439 was not situated in a CpG shore or a CpG island. Therefore, we found that the alleles associated with rapid arsenic metabolism result in the gain of two CpG sites in CpG islands or shores and loss of one CpG site.

We next examined the methylation status of the potential CpG site in rs3740400 C carriers from Argentina by pyrosequencing. An allele-dose increase was found in methylation for the C-allele (comparing individuals of CA or CC genotypes for the rs3740400 allelic site there was significant difference between the groups, p = 0.031; Fig. S1). This result was expected and is an artefact, because for subjects with one CpG (CA) the methylation level would be half of that for subjects with two CpGs (CC). However, we showed with the analysis of rs3740400 that this potential CpG site was differently methylated depending on *AS3MT* genotype, supporting the idea that non-coding SNPs may render DNA methylation targets. Nevertheless, the degree of methylation was very low (average 3%) in the CC carriers, indicating a small effect on *AS3MT* gene expression. Due to the LD within and around *AS3MT* [12], [23], [30], we therefore decided to make a chromosome-region approach and analyzed many CpG sites in 10q24 in relation to *AS3MT* haplotype. We analyzed the association of the whole *AS3MT* haplotype on DNA methylation of *AS3MT* by array analysis (Infinium HumanMethylation 450K BeadChip). In both the Argentinean and Bangladeshi study groups, the *AS3MT* haplotype was strongly associated with the methylation status of *AS3MT* (Table 2; Fig. S2). The strongest association was found for the CpG site cg18534077 in the Argentinean study group where *AS3MT* haplotype was associated with more methylation (*r* = 0.63, p = 10^−6^, adjusted for multiple testing). An opposite pattern was found for cg15744005 and cg08772003, where the haplotype was associated with less methylation (Table 2). Only the associations for cg15744005 remained significant in both populations after adjustments for multiple comparisons. Cg15744005 was situated at enhancer elements (Table S1). *AS3MT* cg18534077 contained a SNP (rs7085104) that could potentially influence base extension (Table 2), although this SNP was situated 27 bp away from the CpG site for cg18534077.

**Table 2:** Statistically significant correlations between *AS3MT* haplotype and DNA methylation in either Argentina or in Bangladesh, as well as descriptions of the DNA methylation sites.

Name	CpG position*	Relation to CpG island†	Gene	Gene name	Enhancer‡	SNP§	Argentina						Bangladesh							
							*r* ¶	p	FDR-adjustedp-value	β∥ Mean	β Min	B Max		*r* ¶	p	FDR-adjusted p-value	B Mean	β Min	B Max	
cg03493300	104813866	*–*	*CNNM2*	Cyclin M2	TRUE	*–*	0.8	<10^−16^	<10^−32^	0.46	0.31	0.59		0.58	10^−12^	10^−7^	0.29	0.14	0.44	
cg09803321	104913480	*–*	*NT5C2*	5′-nucleotidase, cytosolic II	TRUE	rs35436872 (12)	0.75	<10^−16^	<10^−32^	0.65	0.47	0.79		0.23	0.007	0.95	0.68	0.49	0.8	
cg11667387	104835919	Shore	*CNNM2*		NA	rs943037 (0)	−0.88	<10^−16^	<10^−16^	0.71	0.31	0.97		−0.7	<10^−16^	10^−14^	0.92	0.43	1	
cg00035347	104953608	Shore	*NT5C2*		NA	*–*	−0.75	<10^−16^	10^−13^	0.58	0.47	0.73		−0.22	0.01	0.95	0.57	0.29	0.71	
cg23093090	104574429	Shore	*C10orf26*	Chromosome 10 open reading frame 26	TRUE	*–*	−0.66	10^−13^	10^−8^	0.82	0.72	0.88		−0.37	10^−5^	0.92	0.74	0.44	0.88	
cg18534077	104628846	Shore	*AS3MT*	Arsenic (+3 oxidation state) methyltransferase	NA	rs7085104 (27)	0.63	10^−11^	10^−6^	0.79	0.7	0.86		0.29	0.001	0.95	0.53	0.42	0.66	
cg18367433	105149549	*–*	*USMG5*	Up-regulated skeletal muscle growth 5 homolog (mouse)	NA	rs7093039 (2)	−0.59	10^−10^	10^−5^	0.79	0.67	0.87		−0.13	0.13	0.95	0.77	0.61	0.88	
cg07119830	104412306	*–*	*TRIM8*	Tripartite motif containing 8	NA	*–*	−0.57	10^−9^	10^−4^	0.31	0.21	0.49		−0.21	0.018	0.95	0.24	0.11	0.38	
cg15744005	104629667	Shore	*AS3MT*		TRUE	*–*	−0.57	10^−9^	10^−4^	0.31	0.21	0.46		−0.51	10^−9^	10^−4^	0.32	0.19	0.52	
cg23175074	105212490	Shore	*CALHM2*	Calcium homeostasis modulator 2	NA	*–*	−0.49	10^−6^	0.038	0.15	0.1	0.25		−0.14	0.1	0.95	0.18	0.1	0.27	
cg00894378	104680152	Shore	*CNNM2*		NA	*–*	0.48	10^−6^	0.051	0.64	0.53	0.73		0.11	0.22	0.95	0.68	0.53	0.79	
cg08772003	104629869	Shore	*AS3MT*		TRUE	*–*	−0.28	0.006	1	0.45	0.31	0.87		−0.43	10^−7^	0.043	0.46	0.24	0.68	

* According to NCBI Ref. Sequence: NC_000010.10, H sapiens chromosome 10, GRCh37.p5 Primary Assembly [42].

† Situated in a CpG island, shore, or shelf according to Emboss CpGPlot [41].

‡ Enhancer elements were defined according to Illumina’s annotations (http://www.ncbi.nlm.nih.gov/geo/query/acc.cgi?acc=GPL13534).

§ According to http://www.rforge.net/IMA/snpsites.txt. The numbers in parentheses indicate the distance of the SNP from the CpG site.

¶ *r* = Pearson product-moment correlation coefficient.

∥ Beta (β)-values represent the fraction of methylation and hence range from 0 (unmethylated) to 1 (methylated).

Abbreviations: FDR; False Discovery Rate, SNP = Single nucleotide polymorphism.

### Whole-genome Epigenetic Analysis: Correlation of *AS3MT* Haplotype on the DNA Methylation Pattern of Chromosome 10

**Figure 1:**
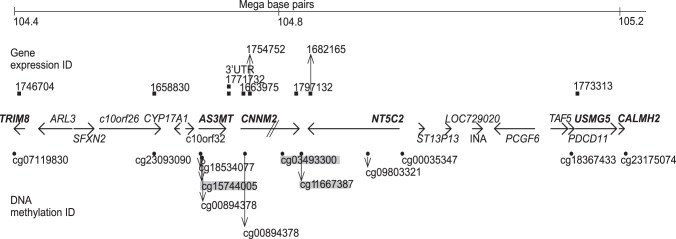
Map of genes in chromosome band 10q24 associated with *AS3MT* haplotype-related DNA methylation. Positions of DNA methylation and gene expression assays are also shown. Black circles denote DNA methylation sites statistically significantly associated with *AS3MT* haplotype in Argentina and the white circle denotes the DNA methylation site statistically significantly associated with *AS3MT* haplotype in Bangladesh. A grey box around the DNA methylation ID denotes DNA methylation sites statistically significantly associated with *AS3MT* haplotype in both Argentina and Bangladesh.

To determine whether adjacent genes are also similarly related to the haplotype, we extended the analysis to an 800 kilobp long region of chromosome 10. In the Argentinean study group, the *AS3MT* haplotype was strongly associated with DNA methylation of genes located 5′ and 3′ of *AS3MT* (a region totalling 800 kilobp around the *AS3MT* gene): *CNNM2*, *NT5C2*, *USMG5*, *TRIM8*, *C10orf26*, and *CALHM2* (in decreasing order of association, Table 2, Fig. 1, and Fig. S2; full gene names are presented in Table 1). Three of these methylation sites were situated at enhancer elements (Table S1). Three of the top twelve methylation sites (*NT5C2* cg09803321, *CNNM2* cg11667387, and *USMG5* cg18367433) contained SNPs that could potentially influence base extension (Table 2). Two of these (*CNNM2* cg11667387 and *USMG5* cg18367433) were situated less than 10 bp from the CpG site.

The *AS3MT* haplotype was associated with more methylation of two sites (one of borderline significance) in *CNMM2* and one site in *NT5C2*. The *AS3MT* haplotype was also associated with less methylation of one site each in *CNNM2*, *NT5C2*, *C10orf26*, *USMG5*, *TRIM8*, and *CALHM2*. There was a similar pattern in the Bangladeshi population, where *AS3MT* haplotype strongly influenced the methylation status of *CNNM2* (Table 2). The directions of associations (i.e., whether the haplotype was associated with more or less methylation of a specific CpG site) were the same in Bangladesh as in Argentina for the other methylation sites, although these associations were not statistically significant in Bangladesh after adjustments for multiple testing.

The DNA methylation of *AS3MT* correlated with the DNA methylation of other genes (5′–3′ direction: *TRIM8*, *C10ORF26*, *CNNM2*, *NT5C2*, *USMG5*, and *CALHM2*) within the 800 kb segment of chromosome 10q24 both in Argentina and in Bangladesh (Table S2). We evaluated if the correlation in DNA methylation of genes around *AS3MT* was particularly strong compared to other regions on chromosome 10, by analyzing windows of 20 consecutive genes and the fraction of CpGs that were correlated between the genes with Pearson correlation >±0.5 (excluding the correlations of CpGs within the same gene). When compared with a permuted (randomized) version of chromosome 10 (green line in plot; Fig. S3) there were some regions that were more correlated than expected. *AS3MT* (dashed red line) seemed to be in a region with average correlation values.

One mechanism of arsenic toxicity may be through alterations in methylation; therefore, we next tested whether arsenic exposure affected DNA methylation in our samples. We found no clear effect of arsenic exposure on DNA methylation in the studied chromosomal region. In the Argentinean study group, arsenic concentrations in the women’s urine were positively correlated with DNA methylation levels of *CALHM2* (*r_s_* = 0.29, p = 0.012), but not with the other methylation sites that were statistically significantly associated with *AS3MT* haplotype. In the Bangladeshi study group, arsenic concentration in maternal urine was inversely correlated with *CNNM2* CpG sites cg11667387 (*r_s_* = −0.23, p = 0.012) and cg00894378 (*r_s_* = −0.21, p = 0.019) in cord blood.

### Influence of *AS3MT* Haplotype on the DNA Methylation Pattern of Chromosome 10: Multivariate Models

CpG sites that were statistically significantly correlated (after adjustments for multiple comparisons) with *AS3MT* haplotype in either Argentina or Bangladesh were further analyzed in univariable and multivariable regression models (Table 3). In total, twelve sites were evaluated; here no further adjustments were made for multiple comparisons. Adjustments were made for total urinary arsenic (natural ln transformed), age and use of coca leaves in Argentina; and for total arsenic in maternal urine (gestational weeks 6–8; natural ln transformed), maternal age and sex of the child in Bangladesh. The results were very similar in the univariable and multivariable models; all sites that were statistically significantly associated with *AS3MT* haplotype in the univariable analyses remained statistically significant in the multivariable analyses, and the β-values remained the same or changed only marginally.

**Table 3:** Univariable and multivariable regression analyses comprising DNA methylation sites that were significantly correlated with *AS3MT* haplotype in either Argentina or in Bangladesh.

Name	Gene	Argentina				Bangladesh			
		β* univariable regression†	p-value univariable regression†	β* multivariable regression‡	p-value multivariable regression‡	β* univariable regression†	p-value univariable regression†	β* multivariable regression§	p-value multivariable regression§
cg03493300	*CNNM2*	0.080	<10^−16^	0.080	<10^−16^	0.052	10^−12^	0.052	10^−12^
cg09803321	*NT5C2*	0.070	<10^−16^	0.070	<10^−16^	0.029	0.007	0.027	0.006
cg11667387	*CNNM2*	−0.33	<10^−16^	−0.33	<10^−16^	−0.12	<10^−16^	−0.12	<10^−16^
cg00035347	*NT5C2*	−0.066	<10^−16^	−0.066	<10^−16^	−0.031	0.01	−0.030	0.01
cg23093090	*C10orf26*	−0.036	10^−13^	−0.036	10^−12^	−0.057	10^−5^	−0.060	10^−5^
cg18534077	*AS3MT*	0.035	10^−11^	0.035	10^−11^	0.026	0.001	0.024	10^−4^
cg18367433	*USMG5*	−0.042	10^−10^	−0.043	10^−10^	−0.010	0.13	−0.013	0.25
cg07119830	*TRIM8*	−0.061	10^−9^	−0.060	10^−9^	−0.020	0.018	−0.021	0.028
cg15744005	*AS3MT*	−0.050	10^−9^	−0.049	10^−9^	−0.062	10^−9^	−0.060	10^−10^
cg23175074	*CALHM2*	−0.021	10^−6^	−0.020	10^−6^	−0.009	0.1	−0.009	0.095
cg00894378	*CNNM2*	0.027	10^−6^	0.028	10^−6^	0.010	0.22	0.011	0.22
cg08772003	*AS3MT*	−0.032	0.006	−0.032	0.006	−0.069	10^−7^	−0.069	10^−7^

* The β–coefficient refers to the slope of the regression line (change in methylation level) with increasing numbers of copies of the haplotype.

† β and p-value from univariable regression. The models look as follows: Methylation level = α+β_1_×copies of *AS3MT* haplotype.

‡ β and p-value from multivariable regression (Argentina). The model look as follows: Methylation level = α+β_1_×copies of *AS3MT* haplotype+β_2_×total urinary arsenic (natural ln transformed)+β_3_×age+β_4_×use of coca leaves (yes/no).

§ β and p-value from multivariable regression (Bangladesh). The model look as follows: Methylation level = α+β_1_×copies of *AS3MT* haplotype+β_2_× total arsenic in maternal urine around gestational weeks 6–8 (natural ln transformed)+β_3_×maternal age+β_4_×sex of child.

### Whole Genome Expression Data: Associations between DNA Methylation Pattern of Chromosome 10 and Gene Expression (Argentina)

**Figure 2:**
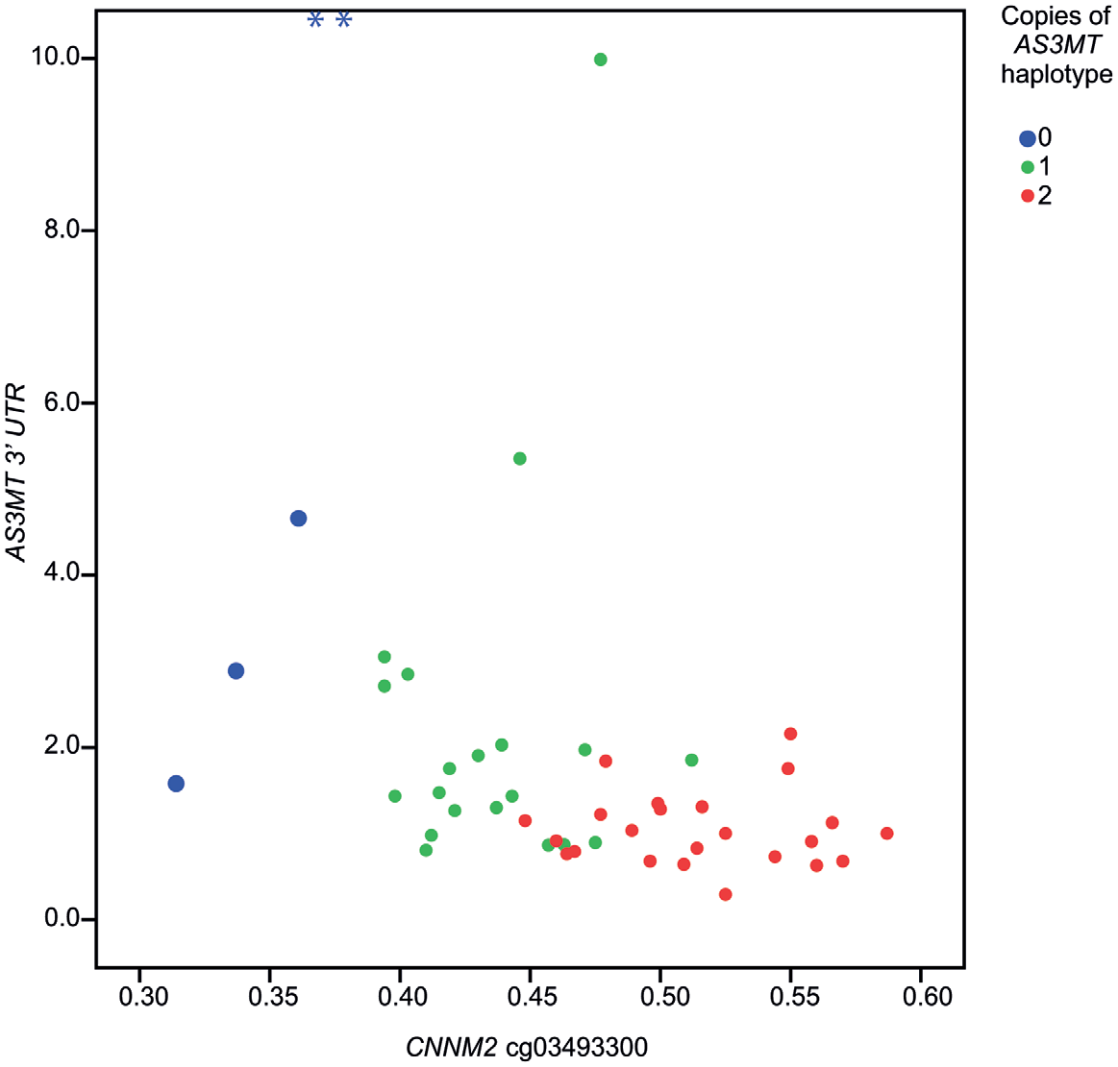
The relationship between *CNNM2* DNA methylation and *AS3MT* gene expression, stratified for *AS3MT* haplotype. Scatterplot depicting the relationship between DNA methylation (expressed as fraction of methylated CpG) for *CNNM2* (X-axis) and gene expression for *AS3MT* (3′UTR; expression relative to the median for individuals with 2 copies of the *AS3MT* haplotype) (Y-axis), where individuals are stratified for *AS3MT* haplotype (total N  = 48). Outliers are denoted by *; the outlier to the left has a relative gene expression of 22.8 and the outlier to the right has a relative gene expression of 24.9.

To determine the effect of the altered DNA methylation in the *AS3MT* haplotype, we also measured expression of *AS3MT* and adjacent genes in our samples from Argentina. The assay of the *AS3MT* 3′untranslated region (UTR), *NT5C2*, *TRIM8* and *USMG5* showed the largest variation in expression between individuals (Table S3). Expression of all genes associated with the *AS3MT* haplotype was correlated with each other (Table S4), but not as strongly as these genes’ DNA methylation was correlated with each other. For the CpG sites positively associated with the *AS3MT* haplotype, inverse correlations (Table 4) were found with gene expression of *AS3MT* (3′UTR assay; p-values≤0.002 or lower for all four CpG sites) and *CNNM2* (p = 0.012 for one CpG site; Fig. 2), whereas positive correlations (Table 4) were found with expression of *NT5C2* (p≤0.014 for three CpG sites) and *USMG5* (p≤0.003 for all four CpG sites, Fig S4). *AS3MT* cg18534077 was associated with both *AS3MT* expression measured with the 3′UTR assay (p = 0.002) and with the *AS3MT* transcript on the array (assay 1771732, p = 0.016).

**Table 4:** Correlations* and effect estimates (β) † between total urinary arsenic (As), DNA methylation and gene expression in Argentina, as well as the influence of haplotype on gene expression.

				Gene expression													
Variable/CpG site		As		*AS3MT* 1771732		*AS3MT* 3′UTR		*CNNM2* 1663975		*CNNM2* 1754752		*CNNM2* 1797132		*NT5C2* 1682165		*USMG5* 1773313	
As	*r_s,_ p-value*	1		−0.20	0.055	0.030	0.83	−0.20	0.062	0.23	0.032	0.12	0.25	−0.004	0.97	0.009	0.93
	β, p-value‡	.		−0.96	0.28	−0.74	0.62	−2.1	0.021	1.1	0.18	0.063	0.92	−12	0.45	0.046	0.99
*AS3MT*	*r_s,_ p-value*	−0.10	0.34	−0.072	0.52	−0.53	<0.001	−.013	0.23	−0.12	0.29	−0.10	0.37	0.21	0.048	0.60	<0.001
haplotype	β, p-value§			−1.5	0.40	−3.8	<0.001	−2.5	0.15	−1.5	0.37	−1.1	0.41	61	0.042	42	<0.001
*A.*																	
*CNNM2*	*r_s,_ p-value*	0.02	0.83	−0.056	0.62	−0.535	<0.001	−0.03	0.79	−0.023	0.84	−0.18	0.11	0.28	0.011	0.58	<0.001
cg03493300	β, p-value¶			−17	0.31	−7.6	<0.001	−2.7	0.87	−9.4	0.56	−14	0.30	890	0.002	400	<0.001
*NT5C2*	*r_s,_ p-value*	−0.03	0.78	0.021	0.85	−0.52	<0.001	−0.22	0.046	−0.033	0.77	−0.099	0.37	−0.031	0.78	0.52	<0.001
cg09803321	β, p-value¶			−14	0.47	−7.2	<0.001	−32	0.10	−11	0.55	−14	0.33	−28	0.93	380	<0.001
*AS3MT*	*r_s,_ p-value*	0.08	0.42	−0.21	0.054	−0.32	0.026	−0.18	0.11	0.025	0.82	−0.059	0.60	0.27	0.014	0.38	<0.001
cg18534077	β, p-value¶			−73	0.016	−11	0.002	−55	0.080	−0.7	0.98	−5.1	0.83	1600	0.002	450	0.001
*CNNM2*	*r_s,_ p-value*	−0.11	0.30	−0.051	0.65	−0.52	<0.001	−0.003	0.98	−0.24	0.032	−0.25	0.023	0.251	0.024	0.32	0.004
cg00894378	β, p-value¶			−34	0.25	−11	<0.001	−1.6	0.96	−66	0.015	−44	0.051	1200	0.014	400	0.003
*B.*																	
*CNNM2*	*r_s,_ p-value*	−0.03	0.78	0.021	0.85	0.56	<0.001	0.14	0.21	0.099	0.38	0.17	0.12	−0.092	0.41	−0.57	<0.001
cg11667387	β, p-value¶			0.73	0.87	1.8	<0.001	7.5	0.095	3.6	0.39	3.2	0.35	−92	0.24	−100	<0.001
*NT5C2*	*r_s,_ p-value*	0.05	0.67	0.048	0.67	0.48	0.001	0.26	0.019	0.10	0.37	0.17	0.12	0.017	0.88	−0.45	<0.001
cg00035347	β, p-value¶			11	0.58	7.7	<0.001	46	0.022	15.2	0.42	22	0.16	−150	0.67	−370	<0.001
*USMG5*	*r_s,_ p-value*	0	0.99	−0.022	0.85	0.39	0.006	0.25	0.024	0.12	0.30	0.21	0.063	−0.18	0.10	−0.67	<0.001
cg18367433	β, p-value¶			−2.4	0.92	7.67	0.003	58	0.015	23	0.31	33	0.068	−860	0.038	−660	<0.001
*AS3MT*	*r_s,_ p-value*	0.16	0.14	0.011	0.92	0.27	0.067	0.14	0.21	0.17	0.12	0.094	0.40	−0.14	0.21	−0.30	0.005
cg15744005	β, p-value¶			11	0.57	4.65	0.060	33	0.097	26	0.15	6.3	0.68	−560	0.096	−270	0.002

A. denotes CpG sites that were positively associated with the *AS3MT* haplotype and B. denotes CpG sites that were inversely associated with the haplotype.

* *r*_s_ and p-value from Spearman correlation.

† β and p-value from multivariable regression.

‡ Model (the β stated in the table is β_1_): Gene expression = α+β_1_× total urinary arsenic (natural ln transformed)+β_2_× RIN.

§ Model (the β stated in the table is β_1_): Gene expression = α+β_1_×copies of *AS3MT* haplotype+β_2_× total urinary arsenic (natural ln transformed)+β_3_×RIN.

¶ Model (the β stated in the table is β_1_): Gene expression = α+β_1_×methylation level+β_2_× total urinary arsenic (natural ln transformed)+β_3_×RIN.

An opposite pattern was observed for the CpG sites inversely associated with the *AS3MT* haplotype (Table 4): positive correlations were found with expression of *AS3MT* (p≤0.003 for three CpG sites) and *CNNM2* (p≤0.022 for three CpG sites), whereas inverse correlations were found with expression of *NT5C2* (p = 0.038 for one CpG site) and *USMG5* (p≤0.002 or lower for all four CpG sites) (Table 4). This was also observed for the CpG sites in *TRIM8*, *C10orf26* and *CALHM2* that were inversely associated with the *AS3MT* haplotype; however, the correlations were less strong (Table S5).

Total arsenic concentration in urine was positively correlated with expression of one transcript of *CNNM2* (1754752, *r_s_* = 0.23), and negatively correlated with the expression of one other transcript of *CNNM2* (1663975, *r_s_* = −0.20) and one transcript of *AS3MT* (1771732, *r_s_* = −0.20) (both of borderline statistical significance, Table 4). Only *CNNM2* 1663975 remained statistically significant in the multivariable analyses (p = 0.021).

Haplotype was associated with gene expression of *AS3MT* 3′UTR (r_s_ = −0.53, p-value<0.001), *NT5C2* (r_s_  = 0.22, p-value = 0.048) and *USMG5* (r_s_  = 0.60, p-value<0.001).

## Discussion

Our results show that the *AS3MT* haplotype, previously associated with a more efficient arsenic metabolism [12], is strongly associated with the methylation status of *AS3MT* and multiple surrounding genes within a large region of 800 kilobp in chromosome band 10q24 in two different populations. In line with the general concept of CpG methylation functioning to reduce gene expression [13], the carriers of the *AS3MT* efficient metabolizing haplotype seemed to have reduced expression of *AS3MT* and *CNNM2.* However, the increased CpG methylation was also associated with higher expression of *NT5C2*, *C10orf26*, *USMG5*, *TRIM8*, and *CALHM2.* Overall, these associations remained stable and statistically significant also after adjustments for other potentially influential variables and show that *AS3MT* haplotype is the major determinant for the methylation and expression pattern in this chromosomal region. Our finding of correlations in DNA methylation between genes around *AS3MT* is not unusual, when we compared to other regions on chromosome 10. However, the strong association of the *AS3MT* haplotype with the DNA methylation pattern, and to some extent to the expression of genes in this region is striking and may reflect the possibility that the genes participate together in arsenic metabolism, or in a not yet characterized pathway.

The strongest association between the *AS3MT* haplotype and DNA methylation of *AS3MT* was found for the CpG site cg18534077, which is located in a CpG shore in the 5′UTR of the gene. We evaluated if the closely located SNP rs3740400 was differentially methylated depending on genotype; however, the methylation levels were very low and this SNP is probably not functional in determining the expression status of *AS3MT*. We previously reported that the SNP rs3740400 is associated with reduced gene expression of *AS3MT* [12], but these data indicate that, despite the fact that rs3740400 creates a CpG site, its effect is probably through linkage with other SNPs in the haplotype. It was unexpected that the *AS3MT* haplotype was associated with reduced *AS3MT* expression, as this haplotype is associated with more proficient arsenic metabolism and more dimethylated arsenic (DMA) in urine [12]. AS3MT is capable of methylating inorganic arsenic to methylarsonic acid (MMA) as well as MMA to DMA [9], [14]. Inorganic arsenic converted into MMA by AS3MT can be released as MMA, undergo a second round of methylation to DMA, or dissociate as MMA bound to glutathione [14]. Why does the low expression of *AS3MT* correlate with increased production of DMA whereas the high expression of *AS3MT* correlates with augmented synthesis of MMA? A possibility is that polymorphisms in other genes linked to the efficient AS3MT haplotype influence the methylation capacity. Clearly, this surprising finding needs to be analyzed further.

The strongest associations with *AS3MT* haplotype and DNA methylation were found for CpG sites related to *CNNM2* (three CpG sites), which in turn were associated with the expression of *AS3MT*, *CNNM2*, *NT5C2*, and *USMG5*. CNNM2 is a magnesium transporter and widely expressed throughout the body. It is mutated in the rare disorder familial hypomagnesia [15] and *CNNM2* SNPs were shown to influence magnesium concentrations in serum [16]. NT5C2 is an enzyme that dephosphorylates non-cyclic nucleoside monophosphates to produce nucleosides and inorganic phosphates [17] and its expression in muscles appears important for regulating energy metabolism and glucose transport [18]. Recently, SNPs in *NT5C2* and the nearby gene *CYP17A1* were found to be associated with fat mass in Japanese women [19]; these SNPs have also been associated with blood pressure [20], [21]. *USMG5* was first recognized as a gene product whose mRNA level increased during skeletal muscle growth in rats [22], and more recently it was shown to be important in maintaining the ATP synthase population in mitochondria indicating that USMG5 is also important in cellular energy metabolism [23]. A recent genome-wide association study of the arsenic metabolism phenotype found SNPs near *AS3MT* to be associated with *USMG5* expression [24].

Furthermore, our data showed that DNA methylation of three other genes (*TRIM8*, *CALHM2*, and *C10orf26*) was associated with the *AS3MT* haplotype. One report shows that *TRIM8* is a direct target of p53 and that TRIM8 induces p53 stabilization and promotes the degradation of MDM2, which in turn directs the p53 response toward growth arrest and not apoptosis [25]. Under stress conditions, p53 promotes transcription of *TRIM8*. CALHM2 belongs to a family of transmembrane glycoproteins that has been suggested to modify calcium homeostasis [26], where three members are closely situated on chromosome 10 [25]. The function of *C10orf26* is unknown. We did not find any association with other closely or more distantly situated genes to *AS3MT*, although several CpG sites per gene were present on the array. Due to the metabolism of arsenic through methylation by the same methyl donor as for DNA methylation, alterations of DNA methylation have been suggested as a toxic mechanism of arsenic [27]. Arsenic exposure *per se* appeared not to strongly modify the methylation or expression of this chromosome region. One speculation is that AS3MT also has other, arsenic-independent, functions in the body and factors related to such a function regulate *AS3MT* expression.

Some methodological aspects need to be commented upon. Firstly, four of the top methylation sites contained SNPs in and around the CpG site, which could bias the results due to impaired base extension since the probe cannot hybridize properly. The influence is stronger the closer the SNP is to the CpG site. Two SNPs (*CNNM2* cg11667387 and *USMG5* cg18367433) were situated less than 10 bp from the CpG site and this warrants caution. However, most of the top methylation sites (eight out of twelve sites), including the top site cg03493300, did not include any SNPs nearby the CpG site.

Secondly, haplotype was treated as a quantitative variable due to the fact that earlier studies of ours [12], [28], [29] have shown a strong allele-dose effect in these populations. The allele-dose effect is also clearly shown in the graphs presented in the supplemental material. In addition, analyses conducted using haplotype as a qualitative variable showed very similar results.

Thirdly, the efficient *AS3MT* haplotype is common in Argentina, but much rarer in Bangladesh. This lack of power in the Bangladeshi population may be a reason to the failure to detect some associations that were present in Argentina but not in Bangladesh.

Fourthly, we have measured DNA methylation in blood, which is a mixture of different cell types with different methylation pattern. We did not have the possibility to sort cells in the blood samples, as this was very difficult to achieve during the field studies. However, we knew from questionnaire data if the study participants had any chronic or acute disease at the time of sampling. These few individuals were excluded from the methylation analysis. Still, the consistency of results between the populations in terms of the associations with haplotype indicates that this likely isn’t a concern in those analyses. We also measured gene expression in blood and not in liver, which is the main metabolizing organ for arsenic, and the DNA methylation and gene expression pattern of *AS3MT* may different in other tissues. Still, there most likely is a similar activity in blood based on the expression (albeit relative low) of the *AS3MT* gene in blood cells [30]. Obtaining tissue samples from individuals in screens of large cohorts is ethically problematic and extremely cumbersome, e.g. if liver biopsies are to be taken and preserved for analysis in the field of the Andes mountains, in contrast to blood sampling, which makes it the method of choice when applicable.

In the region around *AS3MT*, linkage disequilibrium (LD) exists, as noted in Argentina and Bangladesh [12], and the size of the LD block was recently characterized to be at least 350 kilobp in populations in Mexico [31]. A large LD block is present in Asian and European populations as well [10], [31]. One can speculate from the fact that the LD block is widespread in different populations, and that we here show a close relationship between haplotype, gene methylation status and to some extent also gene expression in an even larger region around *AS3MT*, that the LD block contains several genes important for a phenotype, possibly related to arsenic metabolism or to energy metabolism, as suggested from the functional role of several genes in the region.

## Materials and Methods

### Study Populations

#### Main cohort argentina

Participants were women (N = 103) living on the Andean plateau in Northern Argentina and exposed to varying levels of arsenic from their drinking water [32], [33]. Most of the study participants (N = 71) were from the village San Antonio de los Cobres (water arsenic 200 µg/L), with about 5,000 inhabitants, while the remaining participants (N = 32) were from small surrounding villages (with various levels of water As, range 7–73 µg/L). The study individuals were recruited with the assistance of medical personnel, except in the small mining village Tolar Grande, where we went from house to house, explained the project and invited the adults to participate. We included only women, as men were often away from home for work for most of the day, and therefore drank water from different sources. The study area has minimal industrial or traffic pollution. The women in this society rarely drink alcohol or smoke tobacco. 94 women were included in the DNA methylation analyses and 90 women were included in the gene expression analyses, based on availability of high-quality DNA and RNA: 81 women were included in both analyses. Furthermore, 55 of the 103 women were previously analyzed for expression of one transcript in the *AS3MT* gene [12] and these data were included in the analysis of DNA methylation versus gene expression.

#### Bangladeshi cohort

In short, our mother-child cohort in rural Bangladesh is nested in a large, randomized, population-based food and multi-micronutrient supplementation trial, which evaluated nutritional and environmental impacts on pregnancy outcomes and child health [34], [35], [36]. The cohort consists of a sample of 127 women delivering singleton infants at the central Matlab hospital or any of the four connected subcenters during early daytime.

### Ethics Statement

Both verbal and written informed consents were provided by all adult study participants. In Bangladesh, mothers’ gave verbal and written informed consent for use of their cord blood samples. In Argentina, all study subjects gave verbal and written consent, and informed verbal consent was obtained from the next of kin, caretakers, or guardians on the behalf of minors participants involved in the study (N = 4), while the minors participants themselves gave verbal and written consent. The process was documented in special consent forms. The studies, as well as the consent procedure, were approved by the Ministry of Health in Salta, Argentina, the Ethical Review Committee of ICDDR,B, Bangladesh and the Regional Ethical committee at Karolinska Institute, Sweden. The authors complied with all of the legal requirements pertaining to the locations in which the work was done. The data described in our publication are freely available upon request apart from information that will break the confidentiality rules for the study participants.

### Blood and Urine Collection

#### Argentina

Peripheral blood for DNA extraction was collected in K_2_EDTA tubes (Vacuette®, catalogue nr. sc-359548) and blood for RNA extraction was collected in PAX tubes (Beckton Dickinson, catalogue nr. 762165 Franklin Lakes, NJ). Spot urine samples were collected and processed as described previously [37].

#### Bangladesh

Cord blood specimens (mixed arterial and venous) were collected in heparin-coated sterile vials (Becton Dickinson, catalogue nr 367869) at the subcenter health clinics at delivery. Spot urine samples were collected in gestational weeks 6–8 and 30 and analyzed for arsenic.

### Analysis of Arsenic in Blood and Urine

All analyses were performed in the same laboratory. Exposure to inorganic arsenic was assessed by the sum concentration (referred to as total arsenic) of inorganic arsenic, MMA and DMA in urine. Speciation of arsenic metabolites in urine (Argentina) was performed by high performance liquid chromatography (HPLC; Agilent 1100 series system, Agilent Technologies, Germany) coupled with hydride generation and inductively coupled plasma mass spectrometry (ICPMS) (Agilent 7500ce; Agilent Technologies, Japan), employing adequate quality controls [12]. Arsenic in urine from the Bangladeshi women was measured using hydride generation atomic absorption spectrophotometry. The total arsenic concentrations were adjusted to the mean specific gravity (SG) (1.020 g/mL for Argentina and 1.012 for Bangladesh).

### DNA Isolation and Epigenetic Analysis

DNA was isolated using QIAamp® DNA Blood Midi kit (Qiagen, catalogue nr 51183). DNA quality was evaluated on a NanoDrop spectrophotometer (NanoDrop Products,Wilmington, DE) and a Bioanalyzer 2100 (Agilent, Santa Clara, CA) and showed good quality (260/280 nm >1.80). DNA was bisulfite-treated using EZ DNA Methylation kit (Zymo, catalogue nr D5001). The SCIBLU facility (Lund, Sweden) used 200 ng bisulfite-treated DNA for hybridization to the Infinium HumanMethylation 450K BeadChip (lllumina,catalogue nr WG-314-1003). The rs3740400 pyrosequencing assay was evaluated with individuals in Argentina (N = 94). The assay was designed using PyroMark Assay Design 2.0 (Qiagen) software encompassing rs3740400 allelic site with two additional CpG sites. The forward primer was biotinylated. PCR was performed using PyroMark PCR reagents (Qiagen, catalogue nr 972807). The PCR product was purified using Streptavidin Sepharose High Performance beads (Amersham Biosciences, catalogue nr 17-5113-01). Pyrosequencing was done using the PSQ HS96 Pyrosequencing System (Qiagen). The presence of SNPs in probe or query sites was evaluated according to http://www.rforge.net/IMA/snpsites.txt, which contains a list of SNPs in and around the CpG site.

### Genotyping

The *AS3MT* haplotype analyzed is constituted by (5′ to 3′ direction) rs7085104 G, rs3740400 C, rs3740393 C, rs3740390 A, rs11191439 T, rs11191453 C, rs10748835 A, and rs1046778 C. All SNPs, but one (rs11191439), are non-coding. Haplotypes were inferred by PHASE [38]. *Argentina:* Genotyping for these eight SNPs in *AS3MT* was performed in blood DNA using Sequenom technology (Sequenom Inc.,San Diego, CA) [12]. Genotype data was missing for one individual. *Bangladesh:* rs7085104, r3740400, rs3740393, rs3740390 and rs1046778 were genotyped by Taqman allelic discrimination assay (ABI 7900,catalogue nrs. C___3284563_10, C__27510174_10, C__25804287_10, C__27510172_10, C___9596558_10). Genotype frequencies are presented in Table S2. Previous analyses of the LD pattern in another study group from Bangladesh showed that these five SNPs provide a good proxy of the full eight-SNP haplotype [12].

### Gene Expression Analysis

#### Argentina

RNA was extracted using the PAXgene Blood RNA kit (PreAnalytiX, catalogue nr 762174). RNA concentration and purity were evaluated on a NanoDrop spectrophotometer and RNA integrity (RIN) was evaluated on a Bioanalyzer 2100; the results showed good RNA quality (RIN>7.5). DirectHyb HumanHT-12 v4.0 (Illumina, catalogue nr. BD-103-0204) was used for gene expression analysis, at the SCIBLU facility of Lund University. Gene expression data were filtered from background signals with the BioArray Software Environment (BASE) software [39]. In this study, we only evaluated gene expression for the genes that demonstrated an association between *AS3MT* haplotype and DNA methylation pattern. Additionally, we used previously generated data from quantitative real time PCR analysis for expression of *AS3MT* (covering the 3′untranslated region (UTR) (Applied Biosystems Assays-by-design) as further described in Engstrom et al [12]. This was analyzed for 55 individuals; these subjects were matched for age, weight, body mass index (BMI), arsenic in urine, with a more narrow range of arsenic in urine than the individuals included in the HumanHT-12 v4.0 assay (Table 1). Relative gene expression for the *AS3MT* 3′UTR assay was evaluated according to individuals with 2 copies of *AS3MT* haplotype, for which the median gene expression was set to 1 [12].

### Analysis of SNPs or DNA Methylation Sites in CpG Sites, Shores, Shelves and Islands or Enhancers

Whether the SNPs in the *AS3MT* haplotype introduced or removed a CpG site was evaluated by investigating the sequence around the SNP [40]. Whether the SNP or DNA methylation site was situated in a CpG shelf, shore or island was evaluated by using Emboss CpGPlot, which detects CpG-rich areas [40]. Enhancer elements for DNA methylation sites were defined according to annotations by Illumina (GPL-13534, available at http://www.ncbi.nlm.nih.gov/geo/query/acc.cgi?acc=GPL13534).

### Statistical Analysis

Methylation levels are specified by beta (β)-values that represent the fraction of methylation in the sample and range from 0 (unmethylated) to 1 (methylated). β-values were extracted from BeadStudio software (lllumina, San Diego, CA, USA). Missing values were imputed using k-nearest neighbour imputation (k = 10). 0.1% of the values were missing in both Argentina and Bangladesh. Potentially, missing value imputation may increase the correlation in the data. In our case, with so few missing values, this does not shift the data. Principal component analysis (PCA), which capture the major directions of variation in the data, was performed in order to evaluate the influence of technical and biological variables on DNA methylation. For the PCA, we employed the universally applicable singular value decomposition (SVD). The PCA showed that methylation levels were correlated with analysis plate in Bangladesh (two plates were used, while only one plate was used in Argentina). We removed this association from the data by the following procedure: for each methylation site a linear model was fit with the analysis plate as regressor. The residuals of the linear model become the new data for the methylation levels. In this way, the levels of each of the methylation sites were unrelated to analysis plate. No other variables had a major impact on general DNA methylation.

The associations of *AS3MT* haplotype with methylation levels were determined using Pearson correlation (haplotype was used as a numerical variable according to the number of copies, 0–2). Resulting p-values from the 450 K array were corrected for multiple testing (adjusting for 450,000 comparisons) by the Benjamini-Hochberg method to obtain false discovery rates (FDR). In this study we considered FDR <0.05 to indicate significant findings.

The top methylation sites were then further evaluated by building multivariable models. First a univariable model was performed, with each methylation site used separately as a dependent variable, and number of copies of *AS3MT* haplotype was employed as an independent continuous variable. We also evaluated a model using number of copies of *AS3MT* haplotype as a categorical variable; however the results were very similar, indicating an allele-dose effect, and thus haplotype was used as a continuous variable. Multivariable models were built by evaluating, in univariable models, the association between each methylation site and other variables potentially influencing DNA methylation. The variables evaluated in Argentina were urinary arsenic, age (years), BMI, and use of coca leaves, while the variables evaluated in Bangladesh were arsenic measured in maternal urine around gestational weeks 6–8 or 30, birth weight, mothers’ age (years), mothers’ BMI, asset score (similar to socioeconomic status), sex of the baby and gestational age. Variables significantly associated with a methylation site were included in the multivariable models (Table 3). We also evaluated if the haplotype groups were significantly different regarding the potentially influential variables, in order to evaluate stratification according to haplotype (Table 1). This was evaluated with the non-parametric Kruskal-Wallis test.

#### Argentina

The methylation sites that demonstrated a positive association with *AS3MT* haplotype were further analyzed for association with gene expression as well as with total arsenic in urine. The association between *AS3MT* haplotype and gene expression was also. These analyses were evaluated by Spearman’s correlation. Furthermore, a linear regression analysis was performed with gene expression as dependent variable, and methylation level as independent variable, where adjustments were made for total arsenic in urine and RIN. Thereafter, a linear regression analysis was performed, with gene expression as dependent variable, and copies of *AS3MT* haplotype, total arsenic in urine and RIN (to adjust for variations in RNA quality) as independent variables. The statistical analyses were completed by the use of SPSS 18.0 (SPSS Inc, Chicago, IL USA) and R version 2.14.2. Statistical significance refers to P<0.05 (two-tailed).

## Supporting Information

Figure S1 **Influence of** ***AS3MT*** **rs3740400 on DNA methylation. Genotypes CA and CC are presented, since the genotype AA does not carry any potential CpG site.**
(EPS)

Figure S2 **Influence of** ***AS3MT*** **haplotype on DNA methylation of** ***AS3MT*** **,** ***CNNM2*** **and** ***NT5C2*** **.**
(EPS)

Figure S3 **Correlation in DNA methylation between genes around** ***AS3MT*** **compared to other regions on chromosome 10.** This was done by analyzing windows of 20 consecutive genes and the fraction of CpGs that are correlated between the genes with Pearson correlation >±0.5 (excluding CpGs within the same gene). The figure shows a permuted (randomized) version of chr10 (green line in plot) with *AS3MT* indicated (dashed red line).
(EPS)

Figure S4 **The relationship between** ***USMG5*** **DNA methylation and** ***USMG5*** **gene expression, stratified for** ***AS3MT*** **haplotype.**
(EPS)

Table S1 **SNPs and alleles included in the AS3MT haplotype studied.**
(DOCX)

Table S2 **Correlations (Spearman’s rho coefficient) of DNA methylation in genes (5′-3′) in chromosome 10q24 associated with the** ***AS3MT*** **haplotype for A) Argentina and B) Bangladesh.**
(DOCX)

Table S3 **Overview of the gene expression levels for the genes associated with the** ***AS3MT*** **haplotype in Argentina.**
(DOCX)

Table S4 **Correlations in gene expression between genes in chromosome 10q24 (Argentina).**
(DOCX)

Table S5 **Correlations (Spearman ranks) between urinary arsenic (As), DNA methylation (horizontally) and gene expression (vertically) for** ***TRIM8, C10orf26, CALHM2*** **, and** ***USMG5*** **(all CpG sites were negatively associated with the** ***AS3MT*** **haplotype).**
(DOCX)

## References

[1] IARC (2004) Some drinking-water disinfectants and Contaminants, including arsenic: IARC Monographs on the Evaluation of Carcinogenic Risks to Humans. Lyon, International Agency for Research on Cancer; 84. PMC7682301 15645577

[2] NRC (2001) Arsenic in drinking water: 2001 update. Washington DC, National Academy Press.

[3] Ahmed S, Mahabbat-e Khoda S, Rekha RS, Gardner RM, Ameer SS, et al (2011) Arsenic-associated oxidative stress, inflammation, and immune disruption in human placenta and cord blood. Environ Health Perspect 119: 258–264. 20940111 10.1289/ehp.1002086 PMC3040615

[4] Banerjee N, Banerjee S, Sen R, Bandyopadhyay A, Sarma N, et al (2009) Chronic arsenic exposure impairs macrophage functions in the exposed individuals. J Clin Immunol 29: 582–594. 19513820 10.1007/s10875-009-9304-x

[5] Smith AH, Marshall G, Yuan Y, Ferreccio C, Liaw J, et al (2006) Increased mortality from lung cancer and bronchiectasis in young adults after exposure to arsenic in utero and in early childhood. Environ Health Perspect 114: 1293–1296. 16882542 10.1289/ehp.8832 PMC1551995

[6] Vahter M (2002) Mechanisms of arsenic biotransformation. Toxicology 181–182: 211–217. 10.1016/s0300-483x(02)00285-8 12505313

[7] Lindberg AL, Rahman M, Persson LA, Vahter M (2008) The risk of arsenic induced skin lesions in Bangladeshi men and women is affected by arsenic metabolism and the age at first exposure. Toxicol Appl Pharmacol 230: 9–16. 18336856 10.1016/j.taap.2008.02.001

[8] Chung WH, Sung BH, Kim SS, Rhim H, Kuh HJ (2009) Synergistic interaction between tetra-arsenic oxide and paclitaxel in human cancer cells in vitro. Int J Oncol 34: 1669–1679. 19424586

[9] Lin S, Shi Q, Nix FB, Styblo M, Beck MA, et al (2002) A novel S-adenosyl-L-methionine:arsenic(III) methyltransferase from rat liver cytosol. J Biol Chem 277: 10795–10803. 11790780 10.1074/jbc.M110246200

[10] Wood TC, Salavagionne OE, Mukherjee B, Wang L, Klumpp AF, et al (2006) Human arsenic methyltransferase (AS3MT) pharmacogenetics: gene resequencing and functional genomics studies. J Biol Chem 281: 7364–7373. 16407288 10.1074/jbc.M512227200

[11] Vahter M, Concha G, Nermell B, Nilsson R, Dulout F, et al (1995) A unique metabolism of inorganic arsenic in native Andean women. Eur J Pharmacol 293: 455–462. 8748699 10.1016/0926-6917(95)90066-7

[12] Engstrom K, Vahter M, Mlakar SJ, Concha G, Nermell B, et al (2011) Polymorphisms in arsenic(+III oxidation state) methyltransferase (AS3MT) predict gene expression of AS3MT as well as arsenic metabolism. Environ Health Perspect 119: 182–188. 21247820 10.1289/ehp.1002471 PMC3040604

[13] Deaton AM, Bird A (2011) CpG islands and the regulation of transcription. Genes Dev 25: 1010–1022. 21576262 10.1101/gad.2037511 PMC3093116

[14] Marapakala K, Qin J, Rosen BP (2012) Identification of catalytic residues in the As(III) S-adenosylmethionine methyltransferase. Biochemistry 51: 944–951. 22257120 10.1021/bi201500c PMC3431559

[15] Stuiver M, Lainez S, Will C, Terryn S, Gunzel D, et al (2011) CNNM2, encoding a basolateral protein required for renal Mg2+ handling, is mutated in dominant hypomagnesemia. Am J Hum Genet 88: 333–343. 21397062 10.1016/j.ajhg.2011.02.005 PMC3059432

[16] Meyer TE, Verwoert GC, Hwang SJ, Glazer NL, Smith AV, et al.. (2010) Genome-wide association studies of serum magnesium, potassium, and sodium concentrations identify six Loci influencing serum magnesium levels. PLoS Genet 6. 10.1371/journal.pgen.1001045 PMC2916845 20700443

[17] Bianchi V, Spychala J (2003) Mammalian 5′-nucleotidases. J Biol Chem 278: 46195–46198. 12947102 10.1074/jbc.R300032200

[18] Kulkarni SS, Karlsson HK, Szekeres F, Chibalin AV, Krook A, et al (2011) Suppression of 5′-nucleotidase enzymes promotes AMP-activated protein kinase (AMPK) phosphorylation and metabolism in human and mouse skeletal muscle. J Biol Chem 286: 34567–34574. 21873433 10.1074/jbc.M111.268292 PMC3186409

[19] Hotta K, Kitamoto A, Kitamoto T, Mizusawa S, Teranishi H, et al (2012) Genetic variations in the CYP17A1 and NT5C2 genes are associated with a reduction in visceral and subcutaneous fat areas in Japanese women. J Hum Genet 57: 46–51. 22071413 10.1038/jhg.2011.127

[20] Levy D, Ehret GB, Rice K, Verwoert GC, Launer LJ, et al (2009) Genome-wide association study of blood pressure and hypertension. Nat Genet 41: 677–687. 19430479 10.1038/ng.384 PMC2998712

[21] Newton-Cheh C, Johnson T, Gateva V, Tobin MD, Bochud M, et al (2009) Genome-wide association study identifies eight loci associated with blood pressure. Nat Genet 41: 666–676. 19430483 10.1038/ng.361 PMC2891673

[22] Paivarinne H, Kainulainen H (2001) DAPIT, a novel protein down-regulated in insulin-sensitive tissues in streptozotocin-induced diabetes. Acta Diabetol 38: 83–86. 11757806 10.1007/s005920170018

[23] Ohsakaya S, Fujikawa M, Hisabori T, Yoshida M (2011) Knockdown of DAPIT (diabetes-associated protein in insulin-sensitive tissue) results in loss of ATP synthase in mitochondria. J Biol Chem 286: 20292–20296. 21345788 10.1074/jbc.M110.198523 PMC3121504

[24] Pierce BL, Kibriya MG, Tong L, Jasmine F, Argos M, et al (2012) Genome-wide association study identifies chromosome 10q24.32 variants associated with arsenic metabolism and toxicity phenotypes in bangladesh. PLoS Genet 8: e1002522. 22383894 10.1371/journal.pgen.1002522 PMC3285587

[25] Caratozzolo MF, Micale L, Turturo MG, Cornacchia S, Fusco C, et al (2012) TRIM8 modulates p53 activity to dictate cell cycle arrest. Cell Cycle 11: 511–523. 22262183 10.4161/cc.11.3.19008

[26] Dreses-Werringloer U, Lambert JC, Vingtdeux V, Zhao H, Vais H, et al (2008) A polymorphism in CALHM1 influences Ca2+ homeostasis, Abeta levels, and Alzheimer’s disease risk. Cell 133: 1149–1161. 18585350 10.1016/j.cell.2008.05.048 PMC2577842

[27] Ren X, McHale CM, Skibola CF, Smith AH, Smith MT, et al (2011) An emerging role for epigenetic dysregulation in arsenic toxicity and carcinogenesis. Environ Health Perspect 119: 11–19. 20682481 10.1289/ehp.1002114 PMC3018488

[28] Schlawicke Engstrom K, Broberg K, Concha G, Nermell B, Warholm M, et al (2007) Genetic polymorphisms influencing arsenic metabolism: evidence from Argentina. Environ Health Perspect 115: 599–605. 17450230 10.1289/ehp.9734 PMC1852682

[29] Schlawicke Engstrom K, Nermell B, Concha G, Stromberg U, Vahter M, et al (2009) Arsenic metabolism is influenced by polymorphisms in genes involved in one-carbon metabolism and reduction reactions. Mutat Res 667: 4–14. 18682255 10.1016/j.mrfmmm.2008.07.003

[30] Su AI, Wiltshire T, Batalov S, Lapp H, Ching KA, et al (2004) A gene atlas of the mouse and human protein-encoding transcriptomes. Proc Natl Acad Sci U S A 101: 6062–6067. 15075390 10.1073/pnas.0400782101 PMC395923

[31] Gomez-Rubio P, Meza-Montenegro MM, Cantu-Soto E, Klimecki WT (2010) Genetic association between intronic variants in AS3MT and arsenic methylation efficiency is focused on a large linkage disequilibrium cluster in chromosome 10. J Appl Toxicol 30: 260–270. 20014157 10.1002/jat.1492 PMC2862143

[32] Concha G, Broberg K, Grander M, Cardozo A, Palm B, et al (2010) High-level exposure to lithium, boron, cesium, and arsenic via drinking water in the Andes of northern Argentina. Environ Sci Technol 44: 6875–6880. 20701280 10.1021/es1010384

[33] Concha G, Nermell B, Vahter M (2006) Spatial and temporal variations in arsenic exposure via drinking-water in northern Argentina. J Health Popul Nutr 24: 317–326. 17366773 PMC3013252

[34] Raqib R, Ahmed S, Sultana R, Wagatsuma Y, Mondal D, et al (2009) Effects of in utero arsenic exposure on child immunity and morbidity in rural Bangladesh. Toxicol Lett 185: 197–202. 19167470 10.1016/j.toxlet.2009.01.001

[35] Moore SE, Prentice AM, Wagatsuma Y, Fulford AJ, Collinson AC, et al (2009) Early-life nutritional and environmental determinants of thymic size in infants born in rural Bangladesh. Acta Paediatr 98: 1168–1175. 19432828 10.1111/j.1651-2227.2009.01292.x PMC2721967

[36] Tofail F, Vahter M, Hamadani JD, Nermell B, Huda SN, et al (2009) Effect of arsenic exposure during pregnancy on infant development at 7 months in rural Matlab, Bangladesh. Environ Health Perspect 117: 288–293. 19270801 10.1289/ehp.11670 PMC2649233

[37] Concha G, Nermell B, Vahter MV (1998) Metabolism of inorganic arsenic in children with chronic high arsenic exposure in northern Argentina. Environ Health Perspect 106: 355–359. 9618352 10.1289/ehp.98106355 PMC1533000

[38] Stephens M, Donnelly P (2003) A comparison of bayesian methods for haplotype reconstruction from population genotype data. Am J Hum Genet 73: 1162–1169. 14574645 10.1086/379378 PMC1180495

[39] Vallon-Christersson J, Nordborg N, Svensson M, Hakkinen J (2009) BASE–2nd generation software for microarray data management and analysis. BMC Bioinformatics 10: 330. 19822003 10.1186/1471-2105-10-330 PMC2768720

[40] NCBI (2006) SNP Database. Available: http://www.ncbi.nlm.nih.gov/SNP.

[41] Rice P, Longden I, Bleasby A (2000) EMBOSS: the European Molecular Biology Open Software Suite. Trends Genet 16: 276–277. 10827456 10.1016/s0168-9525(00)02024-2

[42] NCBI (2006) National Center for Biotechnology Information Nucleotide Database. Available: http://www.ncbi.nlm.nih.gov/entrez/query.fcgi?db=Nucleotide.

