# Supplementary material for: Efficient Arsenic Metabolism — The AS3MT Haplotype Is Associated with DNA Methylation and Expression of Multiple Genes Around AS3MT
Source: PLoS One. 2013 Jan 14;8(1):e53732. doi: 10.1371/journal.pone.0053732 (PMC3544896; doi:10.1371/journal.pone.0053732)
Supplement: Table S1 — SNPs and alleles included in the AS3MT haplotype studied. (DOCX) [file pone.0053732.s005.docx]

Table S1. SNPs and alleles included in the *AS3MT* haplotype studied.

| SNP ID ^*^ | Chr. 10 position^†^ | Type of SNP^‡^ | Allele | CpG site^§^ | CpG shelf, shore or island^¶^ | Effects on the arsenic metabolism^║^ | Genotype frequency Argentina^**^ | Genotype frequency Bangladesh^**^ |
| --- | --- | --- | --- | --- | --- | --- | --- | --- |
| rs7085104 | 104628873 | 5' near gene, A>G | G | New CpG site | In a CpG shore | Argentina: G-allele lower %iAs, lower %MMA, higher %DMA | 6/41/53 | 54/36/10 |
| rs3740400 | 104629465 | Intron, A>C | C | New CpG site | In a CpG island | Argentina: C-allele lower %iAs, lower %MMA, higher %DMA. Bangladesh: CC lower %MMA | 5/42/53 | 37/41/22 |
| rs3740393 | 104636655 | Intron, C>G | C | - | - | Argentina: G-allele higher %iAs, higher %MMA, lower %DMA. Bangladesh: G-allele higher %iAs, lower %DMA. | 48/44/8 | 4/31/65 |
| rs3740390 | 104638480 | Intron, G>A | A | - | - | Argentina: A-allele lower %iAs, lower %MMA, higher %DMA. Bangladesh: A-allele lower %iAs, higher %DMA. | 8/44/48 | 71/25/4 |
| rs11191439 | 104638723 | Met287Thr, T>C | T | Loss of CpG site | - | Bangladesh: MetThr higher %iAs, lower %DMA | 98/2 | Not genotyped |
| rs11191453 | 104659852 | Intron, T>C | C | - | - | Argentina: C-allele lower %iAs, lower %MMA, higher %DMA. Bangladesh: C-allele lower %iAs, higher %DMA | 8/45/47 | Not genotyped |
| rs10748835 | 104660256 | Intron, A>G | A | - | - | Argentina: G-allele higher %iAs, higher %MMA, lower %DMA. Bangladesh: G-allele higher %MMA, lower %DMA | 51/44/5 | Not genotyped |
| rs1046778 | 104661484 | 3’ UTR, T>C | C | - | - | Argentina: C-allele lower %iAs, lower %MMA, higher %DMA. Bangladesh: C-allele lower %MMA. | 5/46/48 | 43/45/12 |

Abbreviations: DMA; dimethylarsinic arsenic, iAs; inorganic arsenic, MMA; methylarsonic acid, SNP; Single Nucleotide Polymorphism, UTR; untranslated region.

^*^Rs (reference SNP ID)-numbers are from NCBI SNP Database [39].

^†^According to NCBI Reference Sequence: NC_000010.10, Homo sapiens chromosome 10, GRCh37.p5 Primary Assembly [41].

^‡^The ancestral allele, according to the NCBI SNP database [39], is denoted first.

^§^ According to the sequence presented in NCBI SNP database [39].

^¶^ According to Emboss CpGPlot [40].

^║^ According to an Argentinean and a Bangladeshi population from Engström et al [12].

^**^ Presented in the following order: ancestral allele homozygote/heterozygote/other allele homozygote, ancestral allele/other allele.
