# Supplementary material for: Efficient Arsenic Metabolism — The AS3MT Haplotype Is Associated with DNA Methylation and Expression of Multiple Genes Around AS3MT
Source: PLoS One. 2013 Jan 14;8(1):e53732. doi: 10.1371/journal.pone.0053732 (PMC3544896; doi:10.1371/journal.pone.0053732)
Supplement: Table S2 — Correlations (Spearman’s rho coefficient) of DNA methylation in genes (5′-3′) in chromosome 10q24 associated with the AS3MT haplotype for A) Argentina and B) Bangladesh. (DOCX) [file pone.0053732.s006.docx]

Table S2. Correlations (Spearman's rho coefficient) of DNA methylation in genes (5’-3’) in chromosome 10q24 associated with the *AS3MT* haplotype for A) Argentina and B) Bangladesh.

| **A. Argentina** | *TRIM8* cg07119830 | | *C10orf26* cg23093090 | *AS3MT* cg18534077 | *AS3MT* cg15744005 | *AS3MT* cg0877200 | *CNNM2* cg00894378 | *CNNM2* cg03493300 | *CNNM2* cg11667387 | *NT5C2* cg09803321 | *NT5C2* cg00035347 | *USMG5* cg18367433 | *CALHM2* cg23175074 |
| --- | --- | --- | --- | --- | --- | --- | --- | --- | --- | --- | --- | --- | --- |
| *TRIM8* | *r*_s_ | 1.00 | 0.57 | -0.38 | 0.57 | 0.41 | 0.004 | -0.29 | 0.48 | -0.37 | 0.63 | 0.40 | 0.24 |
| cg07119830 | p | . | <0.001 | <0.001 | <0.001 | <0.001 | 0.97 | 0.005 | <0.001 | <0.001 | <0.001 | <0.001 | 0.018 |
| *C10orf26* | *r*_s_ | 0.57 | 1.00 | -0.35 | 0.42 | 0.17 | -0.15 | -0.37 | 0.58 | -0.59 | 0.67 | 0.45 | 0.28 |
| cg23093090 | p | <0.001 | . | <0.001 | <0.001 | 0.10 | 0.14 | <0.001 | <0.001 | <0.001 | <0.001 | <0.001 | 0.007 |
| *AS3MT* | *r*_s_ | -0.38 | -0.35 | 1.00 | -0.41 | -0.28 | 0.29 | 0.55 | -0.52 | 0.60 | -0.49 | -0.28 | -0.23 |
| cg18534077 | p | <0.001 | <0.001 | . | <0.001 | 0.007 | 0.01 | <0.001 | <0.001 | <0.001 | <0.001 | 0.006 | 0.027 |
| *AS3MT* | *r*_s_ | 0.57 | 0.42 | -0.41 | 1.00 | 0.87 | -0.10 | -0.20 | 0.40 | -0.24 | 0.70 | 0.41 | 0.29 |
| cg15744005 | p | <0.001 | <0.001 | <0.001 | . | <0.001 | 0.32 | 0.05 | <0.001 | 0.02 | <0.001 | <0.001 | 0.00 |
| *AS3MT* | *r*_s_ | 0.41 | 0.17 | -0.28 | 0.87 | 1.00 | 0.023 | -0.075 | 0.25 | -0.017 | 0.47 | 0.25 | 0.20 |
| cg08772003 | p | <0.001 | 0.10 | 0.007 | <0.001 | . | 0.82 | 0.47 | 0.013 | 0.87 | <0.001 | 0.013 | 0.053 |
| *CNNM2* | *r*_s_ | 0.004 | -0.15 | 0.29 | -0.10 | 0.023 | 1.00 | 0.52 | -0.48 | 0.39 | -0.25 | -0.38 | -0.36 |
| cg00894378 | p | 0.97 | 0.14 | 0.005 | 0.32 | 0.82 | . | <0.001 | <0.001 | <0.001 | 0.016 | <0.001 | <0.001 |
| *CNNM2* | *r*_s_ | -0.29 | -0.37 | 0.55 | -0.20 | -0.07 | 0.52 | 1.00 | -0.73 | 0.66 | -0.42 | -0.43 | -0.36 |
| cg03493300 | p | 0.01 | <0.001 | <0.001 | 0.05 | 0.47 | <0.001 | . | <0.001 | <0.001 | <0.001 | <0.001 | <0.001 |
| *CNNM2* | *r*_s_ | 0.48 | 0.58 | -0.52 | 0.40 | 0.25 | -0.48 | -0.73 | 1.00 | -0.67 | 0.68 | 0.61 | 0.27 |
| cg11667387 | p | <0.001 | <0.001 | <0.001 | <0.001 | 0.013 | <0.001 | <0.001 | . | <0.001 | <0.001 | <0.001 | 0.01 |
| *NT5C2* | *r*_s_ | -0.37 | -0.59 | 0.60 | -0.24 | -0.02 | 0.39 | 0.66 | -0.67 | 1.00 | -0.56 | -0.38 | -0.37 |
| cg09803321 | p | <0.001 | <0.001 | <0.001 | 0.02 | 0.87 | <0.001 | <0.001 | <0.001 | . | <0.001 | <0.001 | <0.001 |
| *NT5C2* | *r*_s_ | 0.63 | 0.67 | -0.49 | 0.70 | 0.47 | -0.25 | -0.42 | 0.68 | -0.56 | 1.00 | 0.59 | 0.28 |
| cg00035347 | p | <0.001 | <0.001 | <0.001 | <0.001 | <0.001 | 0.02 | <0.001 | <0.001 | <0.001 | . | <0.001 | 0.01 |
| *USMG5* | *r*_s_ | 0.40 | 0.45 | -0.28 | 0.41 | 0.25 | -0.38 | -0.43 | 0.61 | -0.38 | 0.59 | 1.00 | 0.18 |
| cg18367433 | p | <0.001 | <0.001 | 0.01 | <0.001 | 0.01 | <0.001 | <0.001 | <0.001 | <0.001 | <0.001 | . | 0.09 |
| *CALHM2* | *r*_s_ | 0.24 | 0.28 | -0.23 | 0.29 | 0.20 | -0.36 | -0.36 | 0.27 | -0.37 | 0.28 | 0.18 | 1.00 |
| cg23175074 | p | 0.02 | 0.01 | 0.03 | 0.005 | 0.053 | <0.001 | <0.001 | 0.009 | <0.001 | 0.005 | 0.091 | . |
| **B. Bangladesh** |  | |  |  |  |  |  |  |  |  |  |  |  |
| *TRIM8* | *r*_s_ | 1.00 | 0.33 | 0.12 | 0.33 | 0.27 | -0.33 | 0.18 | -0.04 | -0.29 | 0.46 | 0.13 | -0.21 |
| cg07119830 | p |  | <0.001 | 0.19 | <0.001 | 0.003 | <0.001 | 0.038 | 0.68 | 0.001 | <0.001 | 0.15 | 0.018 |
| *C10orf26* | *r*_s_ | 0.33 | 1.00 | 0.042 | 0.63 | 0.46 | -0.13 | 0.09 | 0.24 | -0.08 | 0.05 | 0.25 | 0.02 |
| cg23093090 | p | <0.001 |  | 0.64 | <0.001 | <0.001 | 0.16 | 0.30 | 0.007 | 0.36 | 0.61 | 0.005 | 0.79 |
| *AS3MT* | *r*_s_ | 0.12 | 0.04 | 1.00 | 0.18 | 0.28 | -0.13 | 0.43 | -0.10 | 0.44 | -0.02 | 0.32 | -0.13 |
| cg18534077 | p | 0.19 | 0.64 |  | 0.039 | 0.001 | 0.16 | <0.001 | 0.27 | <0.001 | 0.81 | <0.001 | 0.13 |
| *AS3MT* | *r*_s_ | 0.33 | 0.63 | 0.18 | 1.00 | 0.87 | -0.18 | 0.035 | 0.24 | -0.005 | 0.35 | 0.23 | 0.014 |
| cg15744005 | p | <0.001 | <0.001 | 0.039 |  | <0.001 | 0.048 | 0.70 | 0.007 | 0.95 | <0.001 | 0.008 | 0.88 |
| *AS3MT* | *r*_s_ | 0.27 | 0.46 | 0.28 | 0.87 | 1.00 | -0.12 | 0.015 | 0.24 | 0.084 | 0.27 | 0.21 | 0.055 |
| cg08772003 | p | 0.003 | <0.001 | 0.001 | <0.001 |  | 0.17 | 0.87 | 0.006 | 0.35 | 0.002 | 0.018 | 0.54 |
| *CNNM2* | *r*_s_ | -0.33 | -0.13 | -0.13 | -0.18 | -0.12 | 1.00 | -0.017 | 0.17 | 0.15 | -0.10 | 0.11 | 0.33 |
| cg00894378 | p | <0.001 | 0.16 | 0.16 | 0.048 | 0.17 |  | 0.85 | 0.055 | 0.088 | 0.27 | 0.21 | <0.001 |
| *CNNM2* | *r*_s_ | 0.18 | 0.092 | 0.43 | 0.035 | 0.015 | -0.017 | 1.00 | -0.42 | 0.30 | 0.092 | 0.14 | -0.23 |
| cg03493300 | p | 0.038 | 0.30 | <0.001 | 0.70 | 0.87 | 0.85 |  | <0.001 | 0.001 | 0.30 | 0.12 | 0.009 |
| *CNNM2* | *r*_s_ | -0.037 | 0.24 | -0.10 | 0.24 | 0.24 | 0.17 | -0.42 | 1.00 | -0.045 | 0.12 | 0.32 | 0.24 |
| cg11667387 | p | 0.676 | 0.007 | 0.27 | 0.007 | 0.006 | 0.055 | <0.001 |  | 0.62 | 0.20 | <0.001 | 0.006 |
| *NT5C2* | *r*_s_ | -0.29 | -0.082 | 0.44 | -0.005 | 0.084 | 0.15 | 0.30 | -0.045 | 1.00 | -0.18 | 0.25 | 0.038 |
| cg09803321 | p | 0.001 | 0.36 | <0.001 | 0.95 | 0.35 | 0.09 | 0.001 | 0.62 |  | 0.039 | 0.005 | 0.68 |
| *NT5C2* | *r*_s_ | 0.46 | 0.046 | -0.022 | 0.35 | 0.27 | -0.10 | 0.092 | 0.12 | -0.18 | 1.00 | 0.34 | -0.10 |
| cg00035347 | p | <0.001 | 0.61 | 0.81 | <0.001 | 0.002 | 0.27 | 0.30 | 0.20 | 0.039 |  | <0.001 | 0.25 |
| *USMG5* | *r*_s_ | 0.13 | 0.25 | 0.32 | 0.23 | 0.21 | 0.11 | 0.14 | 0.32 | 0.25 | 0.34 | 1.00 | 0.002 |
| cg18367433 | p | 0.15 | 0.005 | <0.001 | 0.008 | 0.018 | 0.21 | 0.12 | <0.001 | 0.005 | <0.001 |  | 0.99 |
| *CALHM2* | *r*_s_ | -0.21 | 0.024 | -0.13 | 0.014 | 0.055 | 0.33 | -0.23 | 0.24 | 0.038 | -0.103 | 0.002 | 1.000 |
| cg23175074 | p | 0.018 | 0.79 | 0.13 | 0.88 | 0.54 | <0.001 | 0.009 | 0.006 | 0.68 | 0.25 | 0.99 |  |
