# Supplementary material for: Efficient Arsenic Metabolism — The AS3MT Haplotype Is Associated with DNA Methylation and Expression of Multiple Genes Around AS3MT
Source: PLoS One. 2013 Jan 14;8(1):e53732. doi: 10.1371/journal.pone.0053732 (PMC3544896; doi:10.1371/journal.pone.0053732)
Supplement: Table S3 — Overview of the gene expression levels for the genes associated with the AS3MT haplotype in Argentina. (DOCX) [file pone.0053732.s007.docx]

Table S3. Overview of the gene expression levels for the genes associated with the *AS3MT* haplotype in Argentina.

| Gene_probe | N | Mean | Median | Variance | Min | Max |
| --- | --- | --- | --- | --- | --- | --- |
| *AS3MT* ILMN_1771732 | 90 | 118 | 117 | 85 | 100 | 143 |
| *AS3MT* 3’UTR^*^ | 55 | 2.5 | 1.3 | 20 | 0.3 | 25 |
| *C10orf26* ILMN_1658830 | 90 | 179 | 176 | 686 | 116 | 228 |
| *CALMH2* ILMN_1766200 | 90 | 172 | 170 | 460 | 120 | 234 |
| *CNNM2* ILMN_1663975 | 90 | 109 | 108 | 87 | 96 | 148 |
| *CNNM2* ILMN_1754752 | 90 | 98 | 96 | 75 | 85 | 131 |
| *CNNM2* ILMN_1797132 | 90 | 105 | 105 | 49 | 85 | 130 |
| *NT5C2* ILMN_1682165 | 90 | 924 | 899 | 27193 | 648 | 1279 |
| *TRIM8* ILMN_1746704 | 90 | 451 | 442 | 5816 | 301 | 709 |
| *USMG5* ILMN_1773313 | 90 | 167 | 164 | 1876 | 94 | 268 |

All transcripts but *AS3MT* 3’UTR are presented as filtered fluorescence signals from Illumina DirectHyb HumanHT-12 v4.0.

Here, Gene expression is relative to a reference rs1046778 *CC* genotype, for which the median gene expression was set to 1 [12].

UTR = Untranslated region.
