# Supplementary material for: Efficient Arsenic Metabolism — The AS3MT Haplotype Is Associated with DNA Methylation and Expression of Multiple Genes Around AS3MT
Source: PLoS One. 2013 Jan 14;8(1):e53732. doi: 10.1371/journal.pone.0053732 (PMC3544896; doi:10.1371/journal.pone.0053732)
Supplement: Table S4 — Correlations in gene expression between genes in chromosome 10q24 (Argentina). (DOCX) [file pone.0053732.s008.docx]

Table S4. Correlations in gene expression between genes in chromosome 10q24 (Argentina).

|  | | *TRIM8* 1746704 | *C10orf26* 1658830 | *AS3MT* 3'UTR | AS3MT 1771732 | *CNNM2* 1663975 | *CNNM2* 1754752 | *CNNM2* 1797132 | *NT5C2* 1682165 | *USMG5* 1773313 | *CALMH2* 1766200 |
| --- | --- | --- | --- | --- | --- | --- | --- | --- | --- | --- | --- |
| *TRIM8* 1746704 | *r*_s_ | 1.00 | 0.015 | -0.050 | 0.022 | 0.20 | -0.10 | -0.072 | 0.44 | -0.13 | 0.15 |
|  | p | . | 0.89 | 0.74 | 0.84 | 0.053 | 0.35 | 0.50 | <0.001 | 0.21 | 0.16 |
|  | N | 90 | 90 | 45 | 90 | 90 | 90 | 90 | 90 | 90 | 90 |
| *C10orf26* 1658830 | *r*_s_ | 1.00 | 1.00 | 0.11 | -0.043 | -0.022 | -0.058 | 0.24 | 0.19 | 0.10 | -0.11 |
|  | p | 0.89 | . | 0.48 | 0.69 | 0.84 | 0.59 | 0.023 | 0.068 | 0.33 | 0.32 |
|  | N | 90 | 90 | 45 | 90 | 90 | 90 | 90 | 90 | 90 | 90 |
| *AS3MT* 3'UTR | *r*_s_ | 1.00 | 0.11 | 1.00 | 0.16 |  | -0.003 | 0.24 | -0.063 | -0.11 | 0.11 |
|  | p | 0.74 | 0.48 | . | 0.30 | 0.59 | 0.98 | 0.11 | 0.68 | 0.48 | 0.49 |
|  | N | 45 | 45 | 55 | 45 | 45 | 45 | 45 | 45 | 45 | 45 |
| *AS3MT* 1771732 | *r*_s_ | 1.00 | -0.043 | 0.16 | 1.00 | 0.066 | -0.25 | 0.087 | -0.21 | 0.008 | -0.040 |
|  | p | 0.84 | 0.69 | 0.30 | . | 0.54 | 0.018 | 0.42 | 0.052 | 0.94 | 0.71 |
|  | N | 90 | 90 | 45 | 90 | 90 | 90 | 90 | 90 | 90 | 90 |
| *CNNM2* 1663975 | *r*_s_ | 1.00 | -0.022 | 0.082 | 0.066 | 1.00 | -0.046 | -0.057 | 0.15 | -0.12 | 0.13 |
|  | p | 0.053 | 0.84 | 0.59 | 0.54 | . | 0.67 | 0.59 | 0.15 | 0.24 | 0.24 |
|  | N | 90 | 90 | 45 | 90 | 90 | 90 | 90 | 90 | 90 | 90 |
| *CNNM2* 1754752 | *r*_s_ | 1.00 | -0.058 | -0.003 | -0.25 | -0.046 | 1.00 | -0.088 | -0.23 | -0.17 | 0.011 |
|  | p | 0.35 | 0.59 | 0.98 | 0.018 | 0.67 | . | 0.41 | 0.028 | 0.11 | 0.92 |
|  | N | 90 | 90 | 45 | 90 | 90 | 90 | 90 | 90 | 90 | 90 |
| *CNNM2* 1797132 | *r*_s_ | 1.00 | 0.24 | 0.24 | 0.087 | -0.057 | -0.088 | 1.00 | -0.080 | -0.091 | -0.15 |
|  | p | 0.50 | 0.023 | 0.11 | 0.42 | 0.59 | 0.41 | . | 0.45 | 0.39 | 0.16 |
|  | N | 90 | 90 | 45 | 90 | 90 | 90 | 90 | 90 | 90 | 90 |
| *NT5C2* 1682165 | *r*_s_ | 1.00 | 0.19 | -0.063 | -0.21 | 0.15 | -0.23 | -0.080 | 1.00 | 0.17 | 0.17 |
|  | p | <0.001 | 0.068 | 1 | 0.052 | 0.15 | 0.028 | 0.45 | . | 0.11 | 0.11 |
|  | N | 90 | 90 | 45 | 90 | 90 | 90 | 90 | 90 | 90 | 90 |
| *USMG5* 1773313 | *r*_s_ | 1.00 | 0.10 | -0.11 | 0.008 | -0.12 | -0.17 | -0.091 | 0.17 | 1.00 | 0.23 |
|  | p | 0.21 | 0.33 | 0.48 | 0.94 | 0.24 | 0.11 | 0.39 | 0.11 | . | 0.027 |
|  | N | 90 | 90 | 45 | 90 | 90 | 90 | 90 | 90 | 90 | 90 |
| *CALMH2* 1766200 | *r*_s_ | 1.00 | -0.11 | 0.11 | -0.040 | 0.13 | 0.011 | -0.15 | 0.17 | 0.23 | 1.00 |
|  | p | 0.16 | 0.32 | 0.49 | 0.71 | 0.24 | 0.92 | 0.16 | 0.11 | 0.027 | . |
|  | N | 90 | 90 | 45 | 90 | 90 | 90 | 90 | 90 | 90 | 90 |
