# Supplementary material for: Efficient Arsenic Metabolism — The AS3MT Haplotype Is Associated with DNA Methylation and Expression of Multiple Genes Around AS3MT
Source: PLoS One. 2013 Jan 14;8(1):e53732. doi: 10.1371/journal.pone.0053732 (PMC3544896; doi:10.1371/journal.pone.0053732)
Supplement: Table S5 — Correlations (Spearman ranks) between urinary arsenic (As), DNA methylation (horizontally) and gene expression (vertically) for TRIM8, C10orf26, CALHM2 , and USMG5 (all CpG sites were negatively associated with the AS3MT haplotype). (DOCX) [file pone.0053732.s009.docx]

Table S5. Correlations (Spearman ranks) between urinary arsenic (As), DNA methylation (horizontally) and gene expression (vertically) for *TRIM8, C10orf26, CALHM2*, and *USMG5* (all CpG sites were negatively associated with the *AS3MT* haplotype).

|  |  | As | *TRIM8 1746704* | *C10orf26 1658830* | *CALMH2 1766200* | *USMG5 1773313* |
| --- | --- | --- | --- | --- | --- | --- |
| As | *r*_s_ | 1.00 | -0.015 | -0.001 | -0.002 | 0.009 |
|  | p |  | 0.89 | 0.99 | 0.99 | 0.93 |
|  | N | 103 | 90 | 90 | 90 | 90 |
| *TRIM8* | *r*_s_ | 0.10 | -0.14 | 0.019 | 0.10 | 0.231 |
| *cg07119830* | p | 0.34 | 0.21 | 0.87 | 0.36 | 0.038 |
|  | N | 94 | 81 | 81 | 81 | 81 |
| *C10orf26* | *r*_s_ | 0.059 | 0.29 | 0.096 | -0.06 | 0.396 |
| *cg23093090* | p | 0.57 | 0.008 | 0.39 | 0.6 | <0.001 |
|  | N | 94 | 81 | 81 | 81 | 81 |
| *CALHM2* | *r*_s_ | 0.26 | -0.11 | 0.00 | -0.10 | -0.23 |
| *cg23175074* | p | 0.012 | 0.33 | 1.00 | 0.37 | 0.039 |
|  | N | 94 | 81 | 81 | 81 | 81 |
| *USMG5* | *r*_s_ | 0.001 | 0.048 | 0.04 | -0.10 | -0.68 |
| *cg18367433* | p | 0.99 | 0.67 | 0.72 | 0.36 | <0.001 |
|  | N | 94 | 81 | 81 | 81 | 81 |
